# Progress in Medicine

**Published:** 1895-07-13

**Authors:** 


### PROGRESS IN MEDICINE.

### DISEASES OF THE RESPIRATORY SYSTEM.

**Phthisis.**—The methods by which infection is conveyed, and the possibility of prevention, have been the subject of great discussion. G. Sims Woodhead recently pointed out the importance of two localities where bacilli frequently gain entrance to the body which are in health strongly guarded.[1] These are the tonsils and certain portions of the intestinal tract, in both of which masses of adenoid tissue are disposed to prevent invasion. However, even the lungs themselves are often affected only secondarily to disease of these localities and the glands connected with them. The Royal Commission on Tuberculosis have presented their report after five years' study of the question, and find that there is ample evidence of the frequency of tuberculosis among animals used for food, and that such food can and does produce the disease in healthy animals who consume it.[2] The flesh of infected cattle contains tubercular matter less often than the organs, glands, and membranes do, but is frequently contaminated by contact with them during slaughtering. If this contamination has been avoided, and a system of inspection employed under which it is prevented, the meat of animals where there is only localised tuberculosis may be eaten without risk. Indeed, the accidental soiling of the meat by the knife or hands which have been employed for removing the tubercular organs explains the divergent results of previous investigators. Unless the method of diagnosis by injection of tuberculin can be relied upon, there are no satisfactory means of distinguishing the infected cattle from the healthy during life. Milk is, above all, a means of conveying infection in food, but as a rule only when the udder of the cow is diseased, and this is easily recognised during life. If the milk is boiled, its dangerous quality is removed entirely, though cooking is an imperfect protection in the case of diseased meat. These facts were ascertained by most elaborate experiments, and will form a reliable basis for prophylactic measures in the future. Great complaints have arisen among dairymen already of the severe measures advocated for the prevention of tubercular infection, and until the medical profession are completely agreed as to the amount of the danger and the best means of preventing it, legislative measures will be, according to Trumbower, more or less abortive.[3] He arrives, from an American standpoint, at conclusions closely agreeing with those formulated by the English Commission. Of late, in the State of New York, 1,000 cattle have been destroyed as tuberculous by the sanitary authorities; but the results of the tuberculin test were by no means satisfactory, and the powers of the board have now been suspended and a commission appointed to investigate the subject afresh.[4] The frequency of tuberculosis in cattle was shown as long ago as 1892, when 43,400, or 8·2 per cent. of all the beeves slaughtered in Prussia, were found to be affected; but the existence in butter of virulent bacilli has been doubted. Roth, however, found them present in two out of twenty samples.[5] The difficulty of prevention here is considerable. Boiling the milk previous to churning is apt to give an objectionable flavour to the butter, though its keeping powers are greatly improved. On the other hand, we may obtain butter germ free by heating the milk for a long time without letting it reach the boiling point. The importance of infection through the alimentary canal has been discussed also by S. Delépine, who shows that a considerable proportion of the cases of tuberculosis in children are due to it, since, if the mesenteric glands are chiefly or alone the parts affected, the channel of infection must almost certainly have been through the intestine.[6] The paper affords a valuable account of the chief facts which show the danger of tuberculous meat and milk, and materially strengthens the weighty evidence detailed by the Royal Commission. However, a salutary caution against exaggerated views of the danger of aërial infection generally is supplied by Arthur Ransome, who has pointed out that, widespread as the sources of the poison are, an almost "absolute security is given by efficient ventilation night and day, by good drainage, and by an adequate supply of light." So much has been written on the subject of late that a "consumption scare" exists, and phthisical patients are in danger of complete ostracism during the long progress of their disease. In comfortable, well ventilated houses infection is almost unknown, and the writer's experience at the Manchester Consumption Hospital was completely in accord with this view; while in dark, crowded, ill-drained dwellings the frequency of infection is, no doubt, great.[7] The alarmist views of Cornet on the fearful mortality in the nursing orders in Germany through infection were only a half truth. The absence of ventilation was the fact that rendered infection possible. Cornet himself in a recent paper confesses that the bacilli are only dangerous where proper precautions against them are not taken. Even if spittoons partly filled with water only are used, it is rare for dissemination of the spores to take place.[8] Virchow, in the ensuing discussion, was inclined to believe that the enormous decrease of phthisis in Government institutions was largely due to improved general hygiene and diet, though he supported the use of the measures against infection advocated by Cornet. The possibility of disinfecting houses inhabited by tubercular patients has been the subject of an investigation by Ransome and Sheridan Delépine. Their results showed that the various methods of fumigation by sulphur, euchlorine, &c., are practically useless, which confirms the conclusions of Koch and his pupils. On the other hand, the direct application to the walls of a wash containing chlorinated lime of the strength of from 1 in 100 to 1 in 10 is apparently effective.[9] Similarly direct sunlight is a certain and rapid destroyer of tubercle bacilli, and exposure to it for nine hours a thoroughly effective means of disinfecting small articles. R. Crosskey points out that bedding, sheets, and curtains should be disinfected by steam or boiling water, while the excreta should have carbolic or other germicides poured upon them, and the floor and walls during the illness should be moistened before being swept.[10] The difficulties in the way of treating phthisis as a notifiable disease are shown by the account of the work undertaken in New York. The Board of Health began in 1889 by distributing leaflets, and then went on to register all persons suffering from tuberculosis. This was compulsory on all public institutions, and voluntary on the part of private practitioners. Inspectors controlled the cases in tenement houses and hotels, and undertook the purification of the rooms in which they had lived, as well as the bacteriological testing of sputa in suspicious cases. Considerable opposition and frequent evasions were met with, but even the poor have now learnt in many cases to take the necessary precautions without any warning from the officials. Messrs. Biggs and Huddleston notice that a much greater diminution of deaths from tuberculosis has taken place there this year than had ever before occurred.[11]

The value of tobacco smoke as a protective is shown by many statistics collected by C. J. Montgomery.[12] Cholera and influenza have been also checked by it, and the workers in tobacco factories are said to enjoy a remarkable degree of immunity from various pulmonary affections. This is, no doubt, a very comforting doctrine, and it is to be hoped that further investigations will confirm his results.

[1] Lancet. Oct. 27, 1894. [2] B. M. J., April 27, 1895. [3] N. Y. M. J., Dec. 29, 1894. [4] Lancet, Mar. 30, 1895. [5] Am. Med. and Surg. Bullet., Mar., 1895. [6] Med. Chron., May, 1895. [7] Med. Chron., Jan., 1895. [8] Lancet, May 18, 1895. [9] B. M. J., Feb. 16, 1895. [10] Birm. Med. Rev., Feb., 1895. [11] Am. J. Med. Sc., Nov., 1894. [12] B. M. J., Mar. 30, 1895.

